# TuLIP (Tunnelled Line Intraluminal Plasty): An Alternative Technique for Salvaging Haemodialysis Catheter Patency in Fibrin Sheath Formation

**DOI:** 10.1007/s00270-019-02189-7

**Published:** 2019-03-01

**Authors:** R. Ahmed, S. A. Chapman, P. Tantrige, A. Hussain, E. W. Johnston, C. Fang, T. Ammar, D. Y. Huang, C. J. Wilkins, G. Garzillo, G. T. Yusuf

**Affiliations:** Department of Radiology, King’s College Hospital, Denmark Hill, London, SE5 9RS UK

**Keywords:** Fibrin sheath, Haemodialysis catheter, Line stripping

## Abstract

**Background:**

Renal patients with a tunnelled haemodialysis line are at risk of fibrin ‘sheath’ formation which can lead to occlusion. Dysfunctional lines are best treated by catheter exchange with a new subcutaneous tunnel; however, there is a risk of scarring, venous stenosis, potential loss of valuable access as well as the risk of infection.

**Method:**

We report a retrospective review of our experience using tunnelled line intraluminal plasty (TuLIP) in 11 patients over 16 months with fibrin sheath formation on pre-existing tunnelled haemodialysis catheters.

**Result:**

All patients responded well to treatment with median line patency post TuLIP reaching 112 days.

**Conclusion:**

TuLIP may have a role in extending catheter lifespan and delaying more invasive intervention.

## Introduction

Fibrin sheath formation is a well-recognised complication of haemodialysis catheters caused by chronic repetitive trauma to the venous intima. It has been proven in animal models that fibrin sheath formation in relation to haemodialysis catheters can occur as early as 14 days with sheer stress-related wall thickening of the intima and media [1]. At early stages, this may be non-occlusive, progressing eventually to complete occlusion.

Fibrin sheath often manifests as deterioration in the quality of haemodialysis. This is clinically reflected as a failure to aspirate despite being able to flush the catheter or failure of both due to complete occlusion.

Whilst the most efficient solution is over the wire catheter replacement, undesirable factors include new tunnel formation, scarring, infection and potential central venous stenosis. Fibrin stripping with a snare device is the most common alternative but requires puncture of the common femoral vein, discomfort for the patient and potential for intravascular complications.

The technique of inflating an angioplasty balloon within the dialysis catheter over an intraluminal wire for fibrin sheath disruption prior to removal/exchange is well described in the literature [2, 3]. Indeed case reports have been performed inflating a balloon within the catheter lumen to dislodge a fibrin sheath surrounding a line prior to catheter removal [4–8]. We report feasibility of a new technique whereby an angioplasty balloon is inflated within the catheter lumen as a salvage treatment method to improve catheter function for haemodialysis without a need for immediate catheter exchange.

## Materials/Methods

TuLIP is one of several methods used at the authors’ institution in recent years, and ethical approval was waived for this retrospective review. Informed consent was sought in all cases with detailed risks explained.

A haemodialysis catheter was considered dysfunctional when suboptimal dialysis occurred with reduction in flow rates below 300 ml/min [2]. Dialysis specialist nurses document for failure by either flushing and/or aspiration and perform a fibrinolytic lock with urokinase (25,000 units per lumen) [9, 10]. Where this failed to improve catheter function the patients were referred to interventional radiology for line salvage. All patients had the same type of catheter from the same manufacturer (HemoStar, Bard). Ex vivo inflation of an 8-mm balloon in a haemodialysis catheter prior to performing the first case of TuLIP did not result in any macroscopic damage to the catheter.

**Fig. 1:**
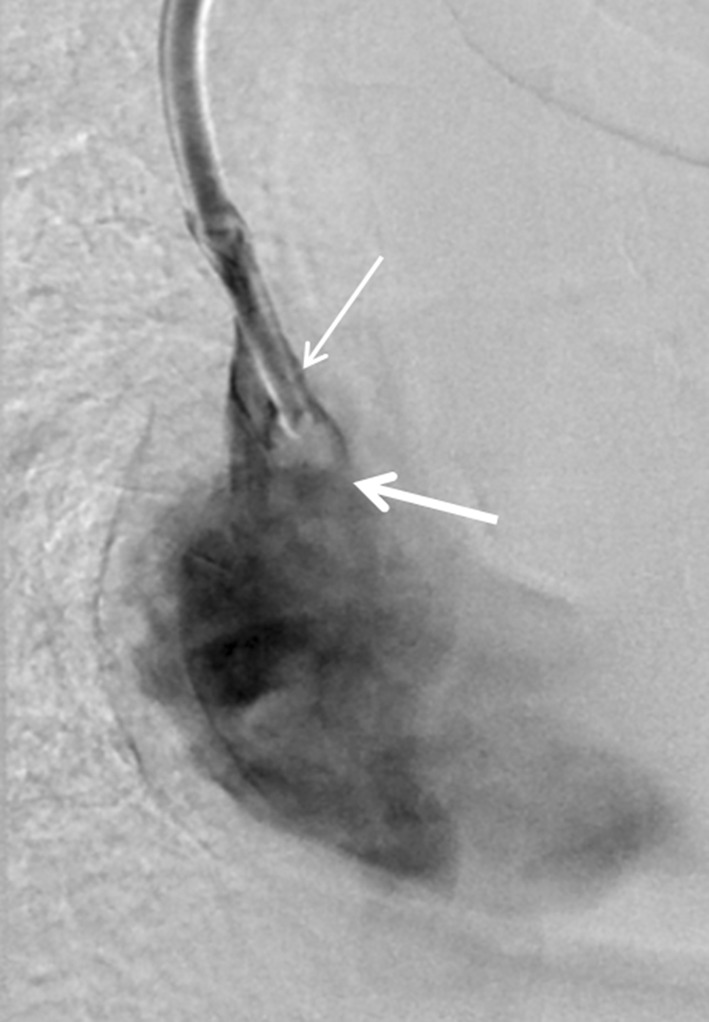
Contrast injection through a haemodialysis catheter in a patient with fibrin sheath formation. A filling defect is seen at the distal tip of the catheter compatible with a small associated thrombus (thick arrow). Contrast is also seen tracking cranially alongside the catheter deep to the fibrin sheath (thin arrow)

All cases were performed by one of four consultant interventional radiologists. In each case, the catheter ports were prepared using chlorhexidine isopropyl alcohol solution or iodopovidone. Aspiration was attempted from both lumens prior to the procedure. Fluoroscopic evaluation was also performed to confirm the presence of a fibrin sheath (Fig. 1). A 0.018″ guidewire (200-300 cm length) was passed through each port of the indwelling catheter and into the inferior vena cava.

Short-shaft (80 cm) and short-length (40 or 60 mm) angioplasty balloons (Bantam, Bard) were used in all cases to assert maximal radial force measuring 5–8 mm in diameter at operator discretion. A larger balloon diameter was used where the operator suspected a lack of success with a smaller size. The balloon was inflated in each lumen sequentially to maximal inflation pressure according to instructions for use (12 atmospheres for the 5-mm balloon and 14 atmospheres for the 6–8-mm balloons). The entire intravascular portion of the line, including the tip, was treated. There were no cases of balloon or catheter rupture fluoroscopically.

**Fig. 2:**
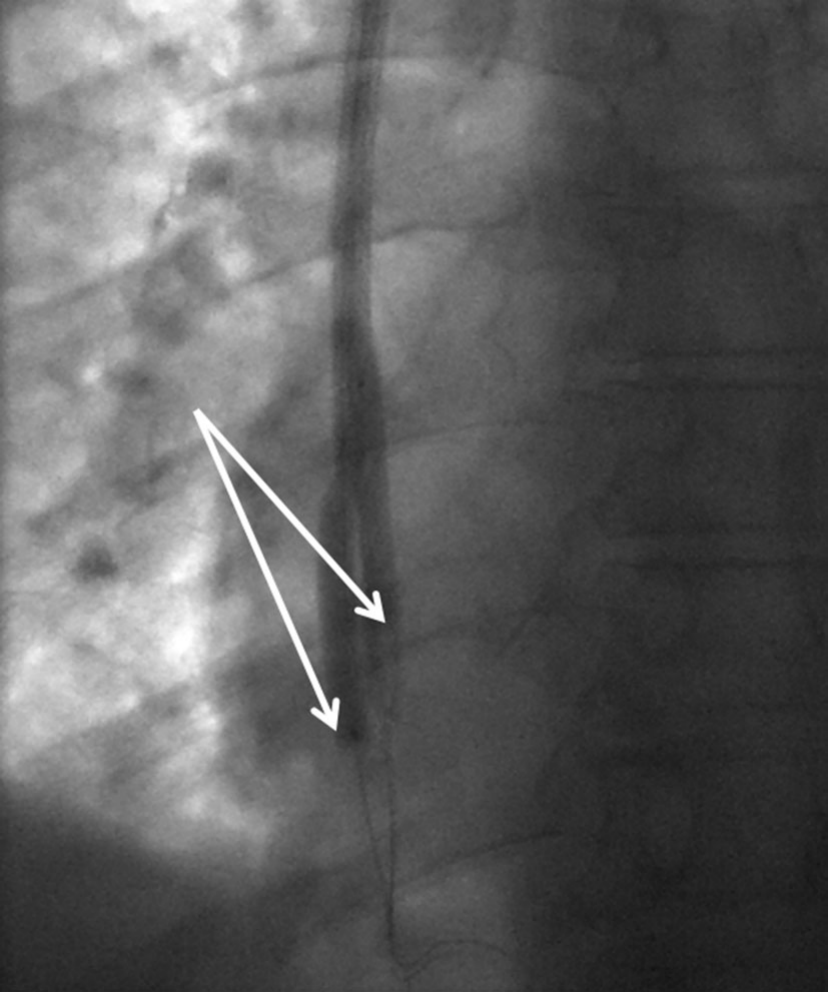
Fluoroscopic image demonstrating over the wire 0.018″ platform standard angioplasty balloons inflated in a ‘kissing’ fashion. Note the balloons are aligned with the offset positions of the distal tips of the venous (distal) and arterial (proximal) lumens (arrows)

In resistant cases, a “kissing TuLIP” technique was used with simultaneous inflation of balloons in both lumens at the same level (Fig. 2).

**Fig. 3:**
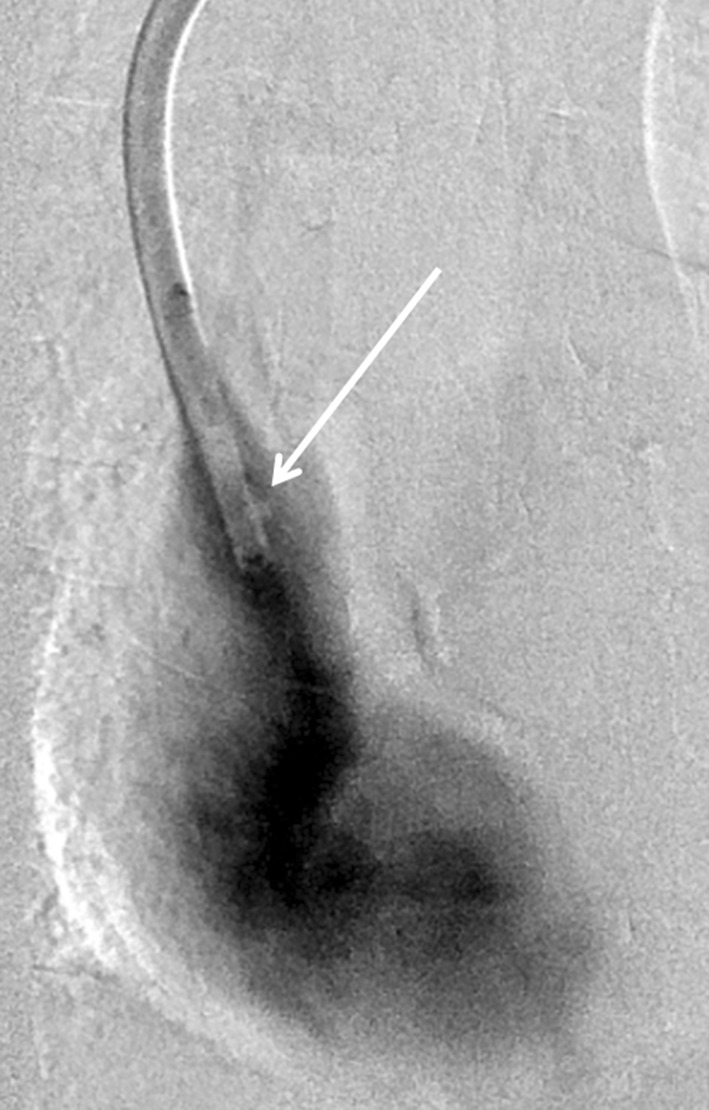
Post-treatment contrast injection demonstrating significant improvement in flow with only a residual filing defect (arrow) alongside the distal tip of the catheter

**Fig. 4:**
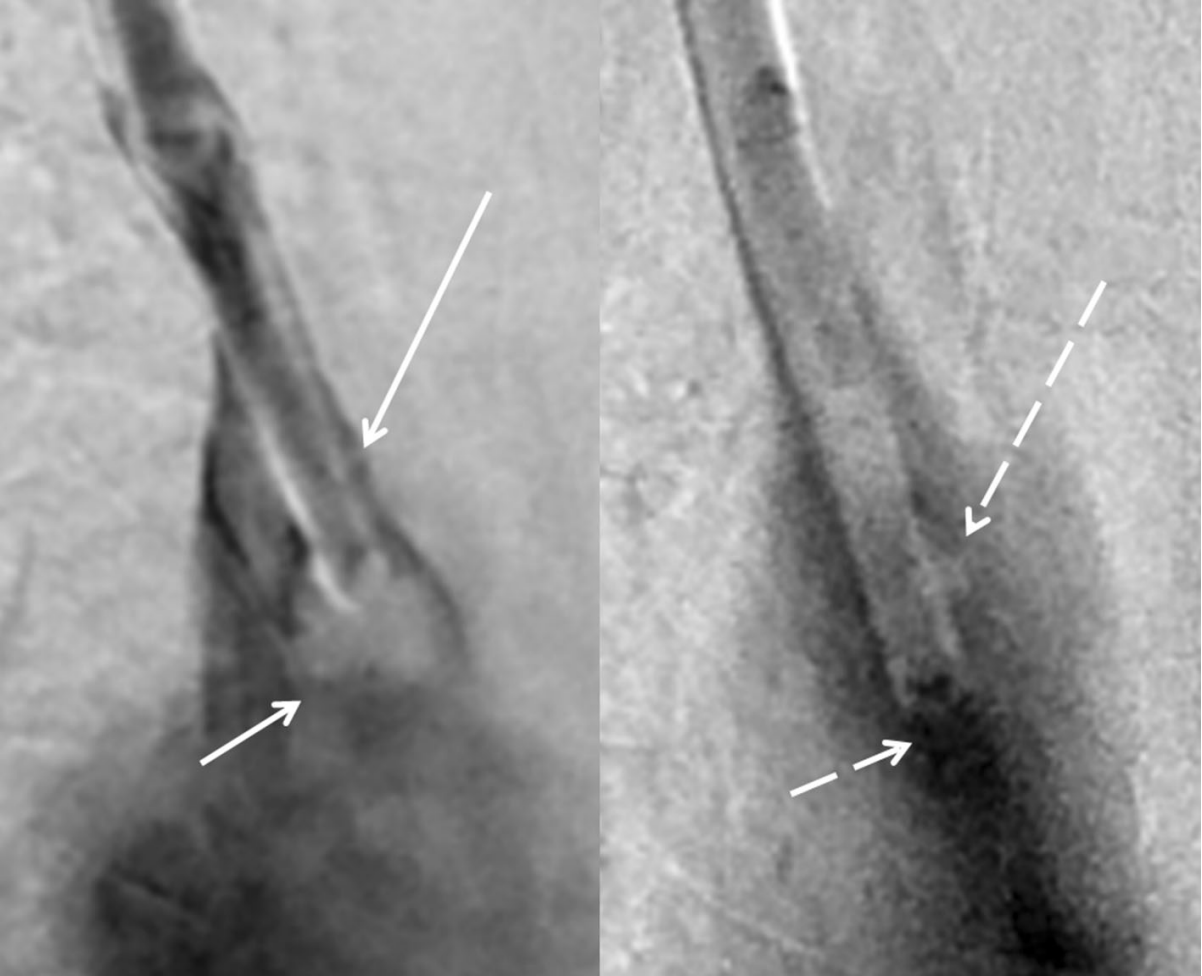
Magnified image from Fig. 1 on the left with pre-procedure contrast injection demonstrating contrast layering under an adherent fibrin sheath (solid long arrow) extending beyond the distal tip of the catheter (solid short arrow). Magnified image from Fig. 3 on the right in the same patient following TuLIP showing disrupted fibrin sheath with only a tiny remnant (long dashed arrow) and greatly improved contrast flow (short dashed arrow)

The procedure was considered successful where free aspiration and flushing without resistance and contrast injection under fluoroscopy without impedance to flow were achieved (Figs. 3, 4). Both lumens of the catheter were then primed and locked with Heparin solution (5000 units/1 ml injected according to the priming volumes stated on the catheter hubs: 1.6–1.8 mls in each lumen). A retrospective review of catheter-related intervention was undertaken using the hospital picture archiving system (PACS) and electronic patient record. The duration of catheter patency has been recorded from date of TuLIP.

## Results

Eleven patients were treated with TuLIP (six male, five female), median age of 65 (49–85 years). In all instances, at least one lumen could be flushed but not aspirated, whilst in some cases, neither lumen could be flushed nor aspirated. All lines had undergone attempted treatment with urokinase.

In total, 10/11 procedures took less than an hour to perform (Table 1). Mean procedural time was 35.3 min (range 18–75 min). All patients demonstrated a positive technical outcome with free flushing and aspiration through both lumens.

**Table 1:** Results following TuLIP

Patient	Age, gender	Procedure time	Length of primary patency	Outcome
A	49, M	75	113 days and ongoing	Continuing haemodialysis
B	71, F	26	112 days	New line insertion
C	65, M	21	33 days	Fistula
D	51, M	31	40 days	Fistula
E	48, F	30	4 days	Fistula
F	65, F	58	190 days	New line insertion
G	69, F	50	180 days	Deceased (unrelated cause)
H	63, M	18	120 days	New line insertion
I	66, M	20	200 days and ongoing	Continuing haemodialysis
J	53, M	19	30 days	Has undergone two conventional line stripping due to residual fibrin sheath
K	85, F	40	21 days	New line insertion

No patient required administration of local anaesthesia or analgesia at the time of procedure as intervention is performed through the catheter lumen. There were no procedure-related complications or complaints of significant pain or discomfort at the time of procedure. Additionally, there were no procedure-related cases of catheter infection. Four cases took longer than 30 min for a combination of reasons including multiple inflations, operator caution and on one occasion, awaiting assistance.

Median length of line patency following TuLIP was 112 days (range 4–200 days). Two patients to date are still successfully dialysing following TuLIP. A further four patients have had catheter replacement following a mean patency of 110 days due to recurrent line dysfunction. Three other patients underwent surgical fistulisation less than two months following TuLIP but maintained dialysis through the treated catheter. One patient had residual fibrin sheath following TuLIP and required two further treatments with conventional stripping, which failed resulting in catheter replacement. One patient passed away of unrelated causes six months after TuLIP without reported line dysfunction in the intervening period.

## Discussion

Our case series demonstrates feasibility for TuLIP to improve a dysfunctional haemodialysis line. In some cases, TuLIP may provide a sufficient temporising measure prior to definitive treatment (e.g. surgical fistula creation) or in those with intractable dialysis line failure in order to avoid recurrent venous access or potential complication of line exchange. The authors recognise that whilst TuLIP may not be suitable for all patients with a failing dialysis catheter, it provides a simple, quick form of treatment to be used for line preservation whilst avoiding catheter exchange or venous access.

Most haemodialysis patients undergo multiple catheter replacements, and efforts should only be made to replace the catheter when all other measures have failed. Venous stenosis, scarring, new tunnel formation, potential loss of venous access as well as patient anxiety are all undesirable factors involved in line replacement. TuLIP may potentially result in fewer catheter exchanges and even potentially negate the need for fistula formation.

The primary limitation of this case series is the small sample size. Additionally, differences in flow rates achieved at haemodialysis pre- and post-procedure were not evaluated. However, prolonged line use after TuLIP suggests procedural success. In cases where there is rapid recurrence of fibrin sheath following TuLIP or incomplete treatment, an alternative technique such as sheath stripping with a snare may be more suitable than a repeat TuLIP but may ultimately require catheter exchange.

A formal prospective randomised control trial is needed to evaluate objective measures of catheter flow rates following TuLIP in comparison with other treatments of a dysfunctional tunnelled line.

## Conclusion

Whilst intraluminal catheter angioplasty is an established method for sheath disruption prior to sheath removal/exchange, our case series demonstrates that TuLIP can be an effective measure to salvage a failing haemodialysis catheter.

## References

[1] Wang LH, Wei F, Jia L, Lu Z, Wang B, Dong HY (2015). Fibrin sheath formation and intimal thickening after catheter placement in dog model: role of hemodynamic wall shear stress. J Vasc Access.

[2] Kennard AL, Walters GD, Jiang SH, Talaulikar GS (2017). Interventions for treating central venous haemodialysis catheter malfunction. Cochrane Database Syst Rev.

[3] Gallieni M, Giordano A, Rossi U, Cariati M (2016). Optimization of dialysis catheter function. J Vasc Access.

[4] Watorek E, Golebiowski T, Letachowicz K, Garcarek J, Kurcz J, Bartosik HA (2012). Balloon angioplasty for disruption of tunnelled dialysis catheter fibrin sheath. J Vasc Access.

[5] Mohamad Ali A, Uhwut E, Liew S (2012). Dialysis catheter fibrin sheath stripping: a useful technique after failed catheter exchange. Biomed Imaging Interv J.

[6] Ni N, Mojibian H, Pollak J, Tal M (2011). Association between disruption of fibrin sheaths using percutaneous transluminal angioplasty balloons and late onset of central venous stenosis. Cardiovasc Intervent Radiol.

[7] Oliver MJ, Mendelssohn DC, Quinn RR, Richardson EP, Rajan DK, Pugash RA (2007). Catheter patency and function after catheter sheath disruption: a pilot study. Clin J Am Soc Nephrol.

[8] d’Othée JB, Tham JC, Sheiman RG (2006). Restoration of patency in failing tunneled hemodialysis catheters: a comparison of catheter exchange, exchange and balloon disruption of the fibrin sheath, and femoral stripping. Vasc Interv Radiol.

[9] Gallieni M, Giordano A, Rossi U, Cariati M (2016). Optimization of dialysis catheter function. J Vasc Access.

[10] Chang DH, Mammadov K, Hickethier T, Borggrefe J, Hellmich M, Maintz D, Kabbasch C (2017). Fibrin sheaths in central venous port catheters: treatment with low-dose, single injection of urokinase on an outpatient basis. Ther Clin Risk Manag.

